# “My dream is to not have to be on a diet”: a qualitative study on burdens of classical homocystinuria (HCU) from the patient perspective

**DOI:** 10.1186/s13023-025-03576-9

**Published:** 2025-03-06

**Authors:** Robin Pokrzywinski, Danaé Bartke, Claudine Clucas, Kathy Machuzak, Lionel Pinto

**Affiliations:** 1 Evidera-PPD, Wilmington, NC USA; 2 HCU Network America, Batavia, IL USA; 3 Evidera-PPD, London, UK; 4 Travere Therapeutics, San Diego, CA USA

**Keywords:** Classical homocystinuria, Cystathionine beta-synthase deficiency, Homocysteine, Homocystinuria, Patient perspective, Qualitative interviews

## Abstract

**Background:**

Patients with classical homocystinuria (HCU) are unable to metabolize homocysteine and rely on dietary treatment to reduce their risk of complications (e.g., thromboembolism, cognitive impairment). Little is known about how patients are affected by their HCU disease experience.

**Methods:**

One-on-one, semi-structured interviews were conducted in adult and pediatric patients (aged ≥ 12 years) with HCU and in primary caregivers on behalf of pediatric patients aged 5–17 years. Interviews elicited patients’ experiences with signs, symptoms, and impacts of HCU. Participants listed their most-bothersome signs/symptoms and impacts and were asked about what changes in HCU treatment would improve their everyday lives.

**Results:**

Eleven adult patients, two pediatric patients, and seven caregivers (of non-participating patients) participated. Many were most bothered by cognition-related symptoms (*n* = 7, 35%) and fatigue (*n* = 6, 30%). Nearly all participants (*n* = 19, 95%) struggled with the “very restricted [low-protein] diet” and the “disgusting” and inconvenient medical formula. The dietary restrictions and requirements often led to challenges fitting in socially. Psychological impacts of HCU (e.g., anxiety, depression) were highly prevalent (*n* = 16, 80%) and bothersome (*n* = 9, 45%). Many patients experienced financial burdens related to their dietary treatment (*n* = 14, 70%). Most participants wanted a treatment involving less formula or a more relaxed diet (*n* = 12, 60%) and felt that these changes would meaningfully improve their everyday lives.

**Conclusions:**

Most patients were burdened by adhering to dietary treatment and by symptoms that worsened when they did not adhere to treatment. These findings can be used to inform treatment goals and care to improve patients’ everyday lives.

**Supplementary Information:**

The online version contains supplementary material available at 10.1186/s13023-025-03576-9.

## Background

Classical homocystinuria (HCU), or homocystinuria due to cystathionine beta-synthase deficiency (MIM: #236200), is a rare genetic disorder characterized by the inability to metabolize homocysteine [1–3]. Patients with untreated HCU can experience multisystem complications [1–4]. Vascular or circulatory complications are common in untreated or insufficiently-controlled HCU and include thromboembolism (e.g., deep vein thrombosis, pulmonary embolism) and stroke [4]. Myocardial infarction can also occur but is less common [4]. In addition, patients can experience neurological complications such as global intellectual impairment [1, 2, 4], psychiatric complications including psychosis and depression [2, 4], and ophthalmic complications like lens subluxation and retinal detachment [4]. Many patients also have bone abnormalities such as osteoporosis or osteopenia, elongated long bones, and scoliosis [1, 2, 4].

Disease severity and age at diagnosis vary widely among patients [4]. Some patients are diagnosed via newborn screening or after seeking medical care during infancy due to difficulties gaining weight or developmental delays [3]. Others are diagnosed during childhood after they experience learning difficulties or ophthalmic complications, such as severe myopia or dislocated lenses [4]. Still others are not diagnosed until adulthood, when they experience a major medical event like thromboembolism [4]. Patients not diagnosed through newborn screening often face long delays between their initial symptoms and HCU diagnosis [5].

Patients generally rely on a restrictive low-protein diet and supplemental formula treatments to prevent the toxic buildup of homocysteine in the blood and thereby reduce the risk of HCU complications [2, 4]. Because many complications of HCU are not immediately apparent, patients need to maintain target homocysteine levels and have their levels checked routinely via blood testing [4]. Treatment with high-dose Vitamin B6 (i.e., pyridoxine) supplements can effectively lower homocysteine levels in some patients with HCU [2, 4], but more than 80% of patients are either completely non-responsive or only partially responsive to Vitamin B6 [6]. Patients who are not completely responsive to Vitamin B6 are advised to adhere to a strict low-protein diet and take a methionine-free medical formula to keep their homocysteine levels within a safe range [2, 4].

Very little is known about how patients with HCU experience their disease and how it impacts their lives. To date, the perspectives of patients with HCU have been reported in only one study – a quantitative survey [5]. In this multinational survey, 80% of patients with HCU (or their caregivers) reported that it was difficult to adhere to the restrictive low-protein diet [5]. In addition, only 50% reported consistently taking their supplemental medical formula, and 26% reported difficulties accessing or paying for treatment [5]. Although surveys can provide important quantitative information about patients’ experiences with HCU, qualitative interviews are needed to help understand these experiences in greater depth.

Insight into patients’ experiences can be used to tailor treatment, care, and drug development to best address patients’ needs. These insights can also be used to develop patient-reported outcome measures that appropriately capture the symptoms and impacts of HCU that matter most to patients. This study aimed to elicit in-depth information about how individual patients are affected by their HCU disease experience, which aspects of HCU they find most bothersome, and what they desire in a future treatment for HCU.

## Methods

### Study design

One-on-one qualitative interviews elicited in-depth perspectives of patients’ experiences with HCU. Interviews were conducted with adult patients, pediatric patients, and caregivers of pediatric patients with HCU. A semi-structured interview guide was developed based on a targeted literature review and feedback from a patient advocate with HCU and three clinicians experienced in treating HCU (See Supplementary methods, Additional File 1). The same interview guide was used for all participants; caregiver participants were asked to respond regarding their observations of their child’s experiences. The study protocol (Pro00064873) was approved by Advarra (Columbia, MD, USA), a central institutional review board. All recruitment procedures complied with current Health Insurance Portability and Accountability Act regulations.

### Participants

Participants were recruited by a patient advocacy group (HCU Network America, Batavia, IL, US) and selected using purposive sampling. Best efforts were made to recruit a diverse sample in terms of sociodemographic and clinical characteristics. Some potential participants were identified from HCU Network America’s patient registry and were invited to participate by email. Other potential participants replied by email to a study advertisement posted on HCU Network America’s social media. Screening was conducted via telephone using a recruitment and screening script.

Eligible participants were adult patients aged ≥ 18 years, pediatric patients aged 12–17 years, or adult primary caregivers of patients aged 5–17 years. Participants self-reported their diagnosis of classical HCU. Participants had to understand, read, and communicate in English; be based in the US; be willing to participate in an audio-recorded interview; and be able to complete study questionnaires autonomously.

Participants provided written informed consent or assent before participating, as did caregivers of participants aged < 18 years. Following participation, participants received a $150 honorarium to compensate them for their time. If a caregiver participated alongside their pediatric patient (aged 12–17 years), each received $150. During screening, four potential participants were excluded because they did not meet eligibility criteria, and two potential participants declined to participate. No participants dropped out after beginning the study.

### Data collection, coding, and analysis

Detailed descriptions of the data collection, coding, and analysis are provided in Supplementary methods, Additional File 1. Interviews were designed to last 90 min, were conducted remotely via telephone or web-based conferencing, and were audio-recorded. First, participants were asked open-ended questions about what they observed that made them feel they (or their child) were having trouble with HCU (i.e., signs of HCU). They were also asked to describe symptoms that they (or their child) have experienced because of HCU and to describe the impacts of HCU on their (or their child’s) daily life. Next, participants were probed to provide further detail about the signs, symptoms, and impacts they reported. Participants were then asked probing questions about signs, symptoms, and impacts that they did not spontaneously mention. Following this, participants were asked to identify the three signs or symptoms (including monitoring concepts) and the three impacts that bothered them (or their child) most. Finally, participants were asked what they would look for in a new treatment for HCU and what would represent a meaningful change. Following the interview, participants completed a brief questionnaire about their (and/or their child’s) sociodemographic and clinical characteristics.

Interviews were transcribed verbatim by a professional transcription vendor (TransPerfect, New York, NY, USA). Transcripts were de-identified and then coded and analyzed iteratively following a thematic approach [7] using ATLAS.ti v9.8 (Scientific Software Development GmbH, Berlin, Germany). Saturation analyses quantified when (i.e., at what point) no substantially new concepts were generated with the analysis of data from additional study participants [8].

## Results

### Participant characteristics

Twenty patients or caregivers participated between August 15, 2022, and October 25, 2022: 11 adult patients, two pediatric patients, and seven caregivers of non-participating pediatric patients (Table 1). Overall, nearly half of patients (9 of 20, 45%) reported that they were diagnosed through a newborn screening program. About one-third (7 of 20, 35%) reported that they were Vitamin B6 responsive. While most patients (16 of 20, 80%) considered their HCU to be mild (6 of 20, 30%) or moderate (10 of 20, 50%) in severity on the day of their interview, several considered their HCU to be severe (3 of 20, 15%) or very severe (1 of 20, 5%).

**Table 1:** Participant demographic and clinical characteristics

Characteristic	Overall (*n* = 20)^a^	Adult patients (*n* = 11)	Pediatric patients (*n* = 2)^b^	Caregivers of pediatric patients (*n* = 7)^c^	
				Caregiver	Patient
Age in years					
Mean (SD)	25.2 (16.1)	37.1 (11.4)	14.0 (1.4)	43.1 (6.0)	9.6 (4.2)
Median (range)	27 (5–65)	33 (24–65)	14 (13–15)	41 (37–50)	9 (5–17)
Gender, female, n (%)	13 (65.0)	8 (72.7)	0 (0.0)	6 (85.7)	5 (71.4)
Race,^d^ n (%)					
White	16 (80.0)	7 (63.6)	2 (100.0)	7 (100.0)	7 (100.0)
Black| African American	2 (10.0)	2 (18.2)	0 (0.0)	0 (0.0)	0 (0.0)
Asian	2 (10.0)	2 (18.2)	0 (0.0)	0 (0.0)	0 (0.0)
Other^e^	3 (15.0)	2 (18.2)	1 (50.0)	0 (0.0)	0 (0.0)
Ethnicity, Hispanic or Latino, n (%)	3 (15.0)	2 (18.2)	0 (0.0)	1 (14.3)	1 (14.3)
Age at diagnosis					
Mean (SD)	7.7 (13.9)	12.1 (17.5)	2.0 (2.8)	n/a	2.4 (4.4)
Median (range)	2.5 (0–54)	5 (0–54)	2 (0–4)	n/a	0 (0–12)
Diagnosed through a newborn screening program, n (%)	9 (45.0)	4 (36.4)	1 (50.0)	n/a	4 (57.1)
Vitamin B6 responsiveness, n (%)					
Responsive	7 (35.0)	5 (45.5)	0 (0.0)	n/a	2 (28.6)
Non-responsive	11 (55.0)	5 (45.5)	2 (100.0)	n/a	4 (57.1)
Not sure	2 (10.0)	1 (9.1)	0 (0.0)	n/a	1 (14.3)
Immediate family member with HCU, n (%)	4 (20.0)	3 (27.3)	0 (0.0)	n/a	1 (14.3)
Current frequency of HCU signs, n (%)					
Never	5 (25.0)	2 (18.2)	0 (0.0)	n/a	3 (42.9)
1–2 days/week	4 (20.0)	1 (9.1)	1 (50.0)	n/a	2 (28.6)
3–4 days/week	2 (10.0)	1 (9.1)	1 (50.0)	n/a	0 (0.0)
Every day	9 (45.0)	7 (63.6)	0 (0.0)	n/a	2 (28.6)
Perceived HCU severity today, n (%)					
Mild	6 (30.0)	3 (27.3)	0 (0.0)	n/a	3 (42.9)
Moderate	10 (50.0)	6 (54.5)	2 (100.0)	n/a	2 (28.6)
Severe	3 (15.0)	2 (18.2)	0 (0.0)	n/a	1 (14.3)
Very severe	1 (5.0)	0 (0.0)	0 (0.0)	n/a	1 (14.3)

Abbreviations: HCU, classical homocystinuria; n/a, not applicable; SD, standard deviation

^a^Overall data describes only the patients represented in the sample: adult patient participants, pediatric patient participants, and the non-participating pediatric patients who were represented by a caregiver

^b^Each of the two pediatric patients (aged 12–17 years) participated alongside one of their caregivers; these caregivers were not included among the seven “Caregiver” participants. The manuscript summarizes data from the patient-caregiver pairs as pediatric patient data. Characteristics reported in the table correspond to the patient

^c^Caregivers responded on behalf of a pediatric patient (aged 5–17 years) for whom they were a primary caregiver; these pediatric patients did not participate in this study

^d^Not mutually exclusive

^e^“Other” races reported: Mexican, Palestine Jewish American

Adult patients had a median age of 33 (range: 24–65) years. Most were female (73%) and White (64%). Adult patients were diagnosed at a median age of 5 (range: 0–54) years; about a third (36%) had been diagnosed through newborn screening. Nearly half (45%) reported being Vitamin B6 responsive.

The two pediatric patients were aged 13 and 15 years. Both were male; one reported being White, and one reported being both White and specifying an “Other” race. One was diagnosed at age 0 years through the newborn screening program, and the other was diagnosed at age 4 years. Neither (0%) reported being Vitamin B6 responsive.

The caregivers of pediatric patients had a median age of 41 (range: 37–50) years and represented patients who had a median age of 9 (range: 5–17) years. Most caregivers and the patients they represented were female (caregivers: 86%; patients: 71%); all (100%) were White. Patients represented by their caregivers were diagnosed at a median age of 0 (range: 0–12) years; more than half (57%) were diagnosed through newborn screening. Less than a third (29%) of these patients were reported to be Vitamin B6 responsive.

### Signs, symptoms, and monitoring of HCU

**Fig. 1:**
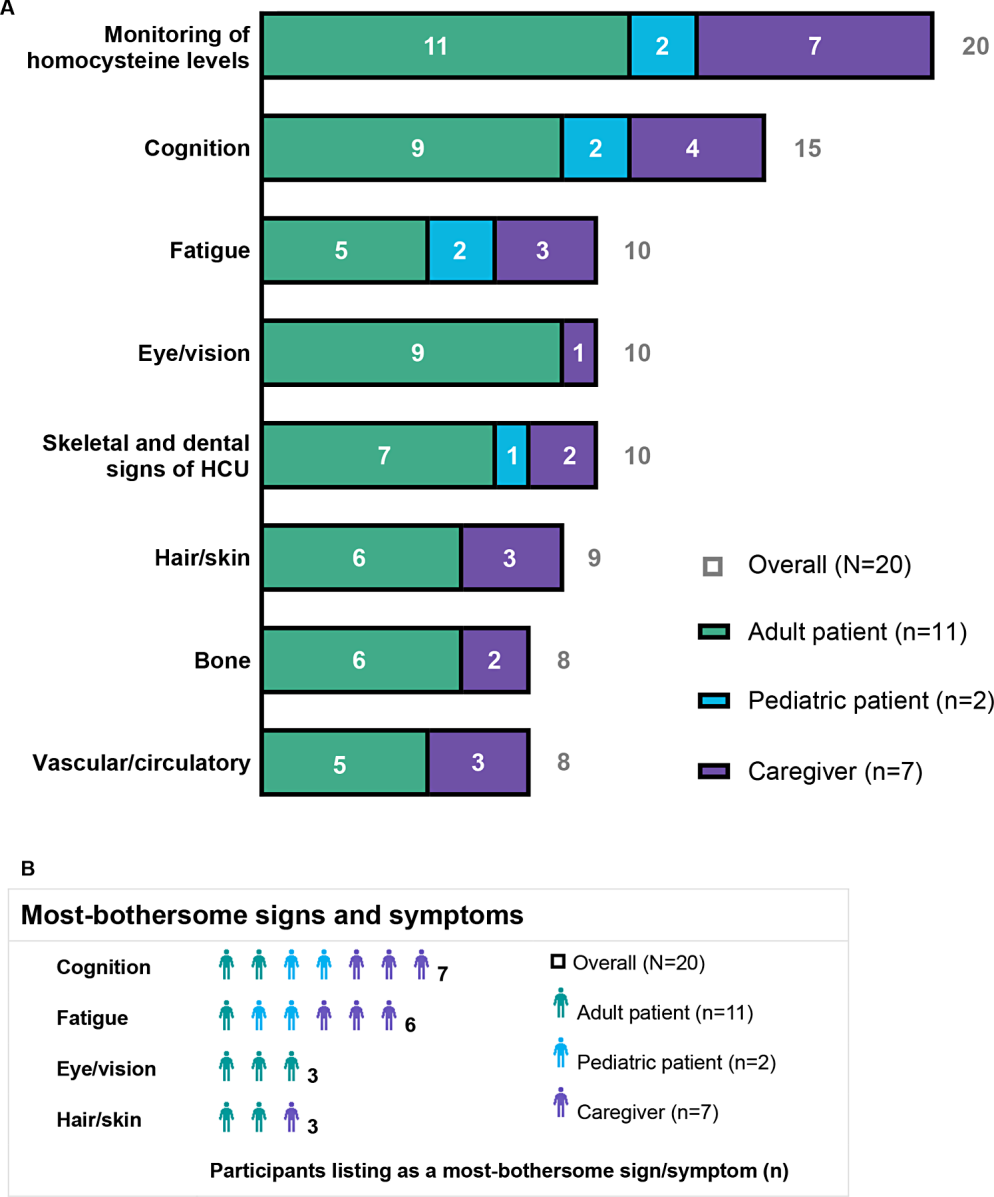
Signs, symptoms, and monitoring of HCU. Signs of HCU, symptoms of HCU, and monitoring of homocysteine levels reported in the overall sample (*N* = 20) and by adult patients (*n* = 11; green), pediatric patients (*n* = 2; blue), and caregivers representing non-participating pediatric patients (*n* = 7; purple). (**A**) Number of participants reporting selected signs of HCU, symptoms of HCU, or monitoring of homocysteine levels. The concepts shown are those reported by ≥ 40% of participants. (**B**) Number of participants listing selected concepts as among their top-three mostbothersome sign-, symptom-, or monitoring-related concepts. The items shown are those reported as among the most bothersome by ≥ 3 participants. Abbreviation: HCU, classical homocystinuria

Participants reported a total of 20 concepts related to their objective observable signs, their subjective symptoms, and monitoring of their HCU (henceforth summarized as “signs or symptoms”; see Supplementary Table 1, Additional File 1). The proportion of the overall sample reporting each sign or symptom and the proportion of the overall sample endorsing the sign or symptom as among their most-bothersome signs and symptoms are depicted in Fig. 1 and described below. These proportions are broken down by participant group (i.e., adult patients, pediatric patients, caregivers of pediatric patients) in Fig. 1, and group-level details are provided in the text when there are notable differences in endorsement of signs and symptoms across participant groups.

All participants discussed monitoring their homocysteine levels (*n* = 20, 100%) (Fig. 1A; Supplementary results, Additional File 1; Supplementary Table 2, Additional File 1). Many signs or symptoms were endorsed by at least 40% of participants: those related to cognition (*n* = 15, 75%), fatigue (*n* = 10, 50%), eyes or vision (*n* = 10, 50%), skeletal and dental signs of HCU (*n* = 10, 50%), hair or skin (*n* = 9, 45%), bones (*n* = 8, 40%), or vascular or circulatory systems (*n* = 8, 40%) (Fig. 1A; Supplementary results, Additional File 1; Supplementary Table 2, Additional File 1). Cognition-related symptoms (*n* = 7, 35%) and fatigue (*n* = 6, 30%) were most frequently reported as among the most-bothersome signs or symptoms, followed by eye or vision issues (*n* = 3, 15%) and hair or skin issues (*n* = 3, 15%) (Fig. 1B).

Participants described getting blood work done to monitor homocysteine levels and detailed changes in their levels over time. Half of patients (10 of 20, 50%) felt that their levels were under control or acceptable, describing their levels as “very good” or “safe” or their doctor as being “very happy”. The other half of patients (10 of 20, 50%) felt that their levels were poorly controlled or high, explaining that their levels were “extremely high”, that their levels have “been elevated for a long time”, or that their levels have “come down, but not to the point where the doctor is comfortable yet”. Participants reported that their levels were affected by adherence to dietary treatment and that high levels impaired their ability to think or speak clearly. Cognition-related symptoms were commonly characterized as brain fog (*n* = 8, 40%), learning disabilities (*n* = 6, 30%), difficulty focusing (*n* = 5, 25%), memory difficulties (*n* = 5, 25%), and difficulty processing information (*n* = 4, 20%). Seven participants (35%) reported cognition-related symptoms as among the most bothersome; most of these participants (5 of 7, 71%) were pediatric patients (2 of 2, 100%) or caregivers of pediatric patients (3 of 7, 43%) (Fig. 1B). Participants described fatigue as “feel[ing] tired”, “laziness”, and having lower energy. Some mentioned “cognitive” fatigue or “mental fatigue […] from just being a HCU patient”. Almost all adult patients (9 of 11, 82%) reported eye or vision symptoms, and three considered these to be among their most-bothersome signs or symptoms (Fig. 1B). Commonly reported eye or vision symptoms included eyesight issues (*n* = 7, 35%), lens dislocation (*n* = 4, 20%), and retinal problems (e.g., retinal detachment) (*n* = 3, 15%). Half of participants (*n* = 10) reported skeletal and dental signs of HCU, which we defined to include differences in skeletal anatomy like tall stature, long limbs and fingers, and dental anomalies. Participants discussed being very tall (*n* = 5, 25%), being thin (*n* = 2, 10%), and having long limbs (*n* = 4, 20%) or long fingers (*n* = 3, 15%). In addition, three participants (15%) reported dental complications, including tooth decay, crooked teeth, and needing dental work. Some patients experienced hair or skin problems (*n* = 9, 45%), such as “thin” hair and “grayish” skin tone; bone-related symptoms (*n* = 8, 40%) like scoliosis (*n* = 5, 25%), broken bones (*n* = 4, 20%), osteoporosis (*n* = 1, 5%), and osteopenia (*n* = 1, 5%); and vascular/circulatory symptoms (*n* = 8, 40%), including blood clots (*n* = 5, 25%), hypertension (*n* = 3, 15%), and other heart-related issues (*n* = 2, 10%). The other signs or symptoms endorsed by ≥ 2 participants were neurological symptoms (*n* = 6, 30%), stomach problems (*n* = 3, 15%), weight gain or obesity (*n* = 2, 10%), low muscle tone (*n* = 2, 10%), respiratory symptoms (*n* = 2, 10%), and pain (*n* = 2, 10%).

### Impacts of HCU

**Fig. 2:**
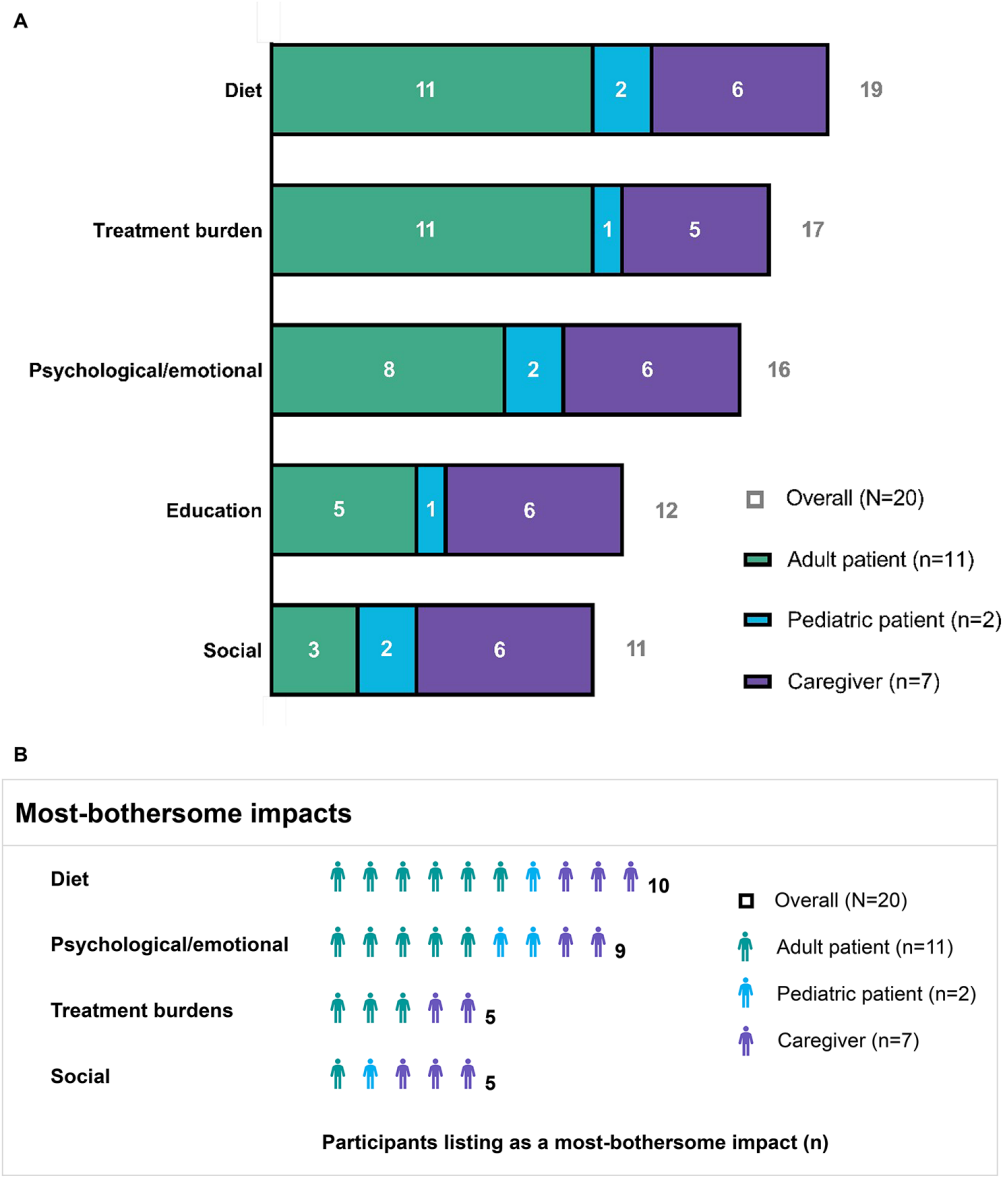
Impacts of HCU on the daily lives of patients. Impacts of HCU on the daily lives of patients, as reported in the overall sample, by adult patients (*n* = 11; green), pediatric patients (*n* = 2; blue), and caregivers representing non-participating pediatric patients (*n* = 7; purple). (**A**) Number of participants reporting selected impacts. The impacts shown are those reported by ≥ 50% of participants. (**B**) Number of participants reporting selected impacts as among their top-three most-bothersome impacts. The concepts shown are reported as among the most bothersome by ≥ 3 participants. Abbreviation: HCU, classical homocystinuria

Participants reported a total of 11 impact-related concepts (see Supplementary Table 3, Additional File 1). The proportion of the overall sample reporting each impact and the proportion of the overall sample endorsing the impact as among their most-bothersome impacts are depicted in Fig. 2 and described below. These proportions are broken down by participant group in Fig. 2, and group-level details are referred to in the text when there are notable differences in endorsement of impacts across participant groups.

Patients’ lives were most commonly affected by dietary restrictions and requirements (*n* = 19, 95%), treatment burdens (*n* = 17, 85%), psychological or emotional impacts (*n* = 16, 80%), educational impacts (*n* = 12, 60%), and social impacts (*n* = 11, 55%) (Fig. 2A; Table 2). The most burdensome impacts were dietary restrictions and requirements (*n* = 10, 50%), psychological or emotional impacts (*n* = 9, 45%), treatment burdens (*n* = 5, 25%), and social impacts (*n* = 5, 25%) (Fig. 2B).

**Table 2:** Representative quotes for the most frequently endorsed impacts

Impact	Example quotes from participant responses
Dietary restrictions and requirements	“It’s a huge impact. […] the stress about it is the fact that everything that you put into that one salad, even if it’s vegetables […] ends up being like, that one meal puts you out for your entire days’ worth of grams of protein, the allotted allowable amounts. Then you’re sitting there, well, I’m still hungry, but I cannot eat any more food.” (Adult patient 300-007-3) “Having to live […] with a very restricted diet and having to drink a terrible formula that’s disgusting. I mean really, that’s the biggest impact that I have.” (Caregiver 300-001-2) “The biggest thing is just the hunger. Just trying to find ways to not always be hungry all the time.” (Adult patient 300-026-3)
Treatment burdens	“[My parents] were having to spend $2000 per month to keep the medication that I needed. They were making payments on that after I had moved out of the house and gone to college and gotten a job.” (Adult patient 300-008-3) “It’s a heavy burden. You can’t just pick up and go somewhere. Everything has to be planned, you know, calculated.” (Caregiver 300-001-2)
Psychological or emotional	“It’s like the depression and the anxiety. I think that’s one of the biggest hurdles for people with this disorder, is accepting it. Because part of the reason I was off diet, […] I didn’t want that constant reminder […] about the fact that I’m constantly different.” (Adult patient 300-24-3) “The most bothersome is the anxiety, just worrying about medical things. […] It’s just the thought that my heart isn’t working properly or that I’m going to have a stroke or some of this because I’m aware of what can happen right here if not treated.” (Adult patient 300-020-3)
Education	“Just being able to focus and keep up, being able to follow multipoint directions independently. […] It might impact his job performance when he just, his brain’s just not working right. He’s just foggy.” (Caregiver 300-022-2)
Social	“She does feel different, […] for example, with her brother, he’s like, so is [patient] ever going to be able to taste meat? Is she ever going to be able to taste fish?” (Caregiver 300-016-2) “It becomes hard for her to speak up in a group or with her peers, because I think she’s worried she’s not going to be able to remember words.” (Caregiver 300-012-2)

### Dietary restrictions and requirements

Nearly all participants reported challenges related to dietary restrictions and supplementation requirements (*n* = 19, 95%) (Fig. 2A; Table 2). Diet-related challenges were the most commonly reported top-three most-bothersome impact, being listed among the most-bothersome impacts for half of the participants (*n* = 10, 50%), including about half of the participants in each group (i.e., adult patients, pediatric patients, and caregivers) (Fig. 2B).

Many participants discussed difficulties following the strict dietary restrictions (*n* = 9, 45%), particularly the limited allotment of natural protein. Several participants described being hungry “all the time” or being hungry but unable to eat more because they had already reached their daily protein allotment. Many explained that they were unable to participate in social activities, like eating birthday cake, while others expressed challenges with restricted food options when going out to eat. One caregiver explained that their child “can really only have French fries or maybe a salad”. Participants also described the extra efforts required to plan the appropriate kinds and quantities of foods that they could eat.

Some described difficulties taking the methionine-free medical formula because of its bad taste and inconvenience to use (*n* = 8, 40%). Participants characterized the taste of the formula as “disgusting”, “terrible”, “gross”, “really, really bad”, and “like you’re licking the bottom of a gym shoe”. Some participants explained that the formula is “acidic” and “make[s] you feel sick”. Patients found it inconvenient to take the formula at work, when traveling, or when going out for the afternoon. In addition, many participants commented on the high costs of supplements and medical foods or their difficulties accessing these products. Finally, eight participants (40%) described taking vitamin supplements to help them manage their HCU. The vitamins and other supplements discussed were folic acid (*n* = 5), vitamin B6 (*n* = 3), vitamin B12 (*n* = 3), vitamin B (*n* = 3), vitamin D (*n* = 2), calcium (*n* = 2), vitamin C (*n* = 1), fish oil (*n* = 1), and multivitamins (*n* = 1).

### Treatment burdens

Almost all participants reported treatment-related burdens (*n* = 17, 85%) (Fig. 2A; Table 2); these burdens were among the most-bothersome impacts for several adult patients and caregivers (Fig. 2B).

Most participants experienced financial burdens related to buying supplemental formula and medical foods (*n* = 14, 70%). Participants explained that the medical foods are “really, really expensive”. Two participants detailed how they had to pay $10 or more for a box of pasta, and one mentioned having to pay additional costs for shipping. Some participants discussed how their health insurance did not cover expenses for the supplemental medical formula and medical foods, or that they were fighting with their insurance companies to gain coverage. Another did not eat medical foods because they were not covered by their insurance. One adult patient explained that even when their insurance covers most treatment costs, specialty food costs add up and become a burden.

Several participants expressed that drinking the formula imposed a psychological burden because it tastes bad and is difficult to tolerate (*n* = 4, 20%). Some participants also described psychological burdens related to the inconveniences of dietary and supplement treatment, including burdens of planning appropriate foods to eat and carrying enough formula and keeping it cold.

### Psychological/emotional

80% of participants (*n* = 16) reported psychological or emotional impacts (e.g., anxiety, depression) related to HCU (Fig. 2A; Table 2). These impacts were among the most-bothersome impacts for nearly half of participants (*n* = 9, 45%), including participants in all groups (Fig. 2B).

Most patients experienced anxiety because of their HCU (*n* = 12, 60%). Many worried about following the diet and about their current or past physical symptoms and described “paranoia” about symptoms worsening and about becoming ill in the future. Two adult patients worried about how childbearing might affect their HCU, for instance due to increased homocysteine levels during or after pregnancy. Some patients experienced depression because of their HCU (*n* = 6, 30%), and some felt frustrated or angry about being on a restrictive diet or about their condition overall (*n* = 6, 30%).

### Education

Education-related impacts were reported by most participants (*n* = 12, 60%), especially caregivers (6 of 7, 86%) (Fig. 2A; Table 2). Only one participant (5%) – an adult patient – considered educational impacts as among their most-bothersome impacts.

Most participants described struggles at school due to “fogginess” or difficulty focusing. Several explained that they had to “work a lot harder”. Some patients needed special accommodations at school (*n* = 6, 30%), such as a tutor or extended time for exams, and some had difficulty finding words or experienced other learning difficulties.

### Social

Many patients experienced social impacts related to HCU (*n* = 11, 55%) (Fig. 2A; Table 2). Social impacts were reported by both pediatric patients (100%) and nearly all (6 of 7, 86%) caregivers, but only 3 of 11 (27%) adult patients. Social impacts were among the most-bothersome impacts for five participants (25%), four (80%) of whom were pediatric patients (1 of 2, 50%) or caregivers representing pediatric patients (3 of 7, 43%) (Fig. 2B). Dietary restrictions made it difficult for patients to fit in when eating with peers or talking about food. Patients “feel different”, cannot join in eating “all these other snacks [they] can’t have” at birthday parties, or cannot respond when asked how certain foods taste. In addition, some patients had challenges socializing because they can’t “say what [they] need to say” or because they are “worried [they’re] not going to be able to remember words”.

### Physical activities

Six participants (30%) reported that HCU impacted their physical activities. Several described not having the stamina to go on walks, not “keep[ing] up with everyone” when playing sports, and having difficulties going up and down stairs. Others reported a fear of falling because of their osteopenia (i.e., low bone density) or being told that they cannot play a sport because their “bones are going to break”.

### Psychiatric

Four participants (20%) reported psychiatric impacts of HCU. One of nine adult patients (11%) and 1 of 2 pediatric patients (50%) had been diagnosed with attention deficit disorder or attention deficit hyperactivity disorder, while 2 of 7 caregivers (29%) reported that their child displayed behaviors of attention deficit disorder or attention deficit hyperactivity disorder but had not been diagnosed.

### Daily activities

Three participants (15%) – all adult patients – reported impacts of HCU on their daily activities. Each described difficulties related to eyesight, including trouble seeing “really small print”, “seeing the beginning […] [or end] of sentences”, or “trouble seeing […] if it’s really bright or […] really dark outside”.

### Limited functioning

Two participants (10%) – both caregivers – described “trouble with organization” or trouble completing multistep tasks such as “washing [their] clothes and hanging them up”.

### Desired changes in new treatment for HCU

Most participants (*n* = 12, 60%) wanted a treatment for their HCU that would involve less formula and a more relaxed diet (Table 3). This desire was shared across participant groups (7 of 11 adult patients, 64%; both pediatric patients, 100%; 3 of 7 caregivers, 43%). Many participants expressed that it would be “relieving” or a “dream” to be able to eat more food, have more food options, or eat more natural protein. Several mentioned that they would appreciate even minor adjustments to the dietary requirements, such as being able to have “even two grams more [of protein]”. Some participants would prefer to change the mode of treatment administration from formula to a pill because it would alleviate the burdens of bad taste or be easier to carry around. Many reported that making the diet more manageable would be a meaningful change and make a difference in their everyday lives.

**Table 3:** Representative quotes about desired new treatments

Example quotes from participant responses
“If they could have a less-restricted diet, that would be amazing. […] they could just fit in more with their peers and eat food a lot more similar to what they’re eating and have options at a restaurant besides French fries. […]. So that they could enjoy being a kid.” (Caregiver 300-012-2)
“That’s like my dream is to not have to be on a diet. Not have to take formula, you know, or have a once-a-week injection or once a month, or twice a week. […] If I couldn’t come off of the diet totally, if it doubled my protein intake or you know halved the amount of formula that I had to take, I would consider that a worthwhile improvement.” (Adult patient 300-008-3)
“I guess if I had more leeway in my diet, it would be a little more relieving. […] It would obviously make a difference because the less grams of protein that you’re allotted, the more strict you have to be. But if you can offset that and say, even two grams more, […] it doesn’t seem like a lot, but it is a lot, could help alleviate some of those dietary restrictions.” (Adult patient 300-005-3)
“More energy, weight gain. […] Happier. […] Fewer days of fatigue, definitely. But also not having to be on a diet.” (Pediatric patient 300-013-1)

Several participants (*n* = 5, 25%), including 2 of 11 adult patients (18%), 1 of 2 pediatric patients (50%), and 2 of 7 caregivers (29%), reported that they wanted a treatment that would improve HCU symptoms, including fatigue (*n* = 3, 15%), cognition-related symptoms (*n* = 2, 10%), eyesight (*n* = 2, 10%), scoliosis (*n* = 1, 5%), and organ-related symptoms (*n* = 1, 5%).

## Discussion

To our knowledge, this is the first study to elicit in-depth information on the patient experience in HCU using qualitative interviews. In this study, 20 US-based patients or caregivers of patients with HCU were guided in discussing various aspects of their or their child’s HCU disease experience. Nearly all participants reported challenges with adhering to a strict low-protein diet, financial and psychological burdens related to HCU dietary restrictions and formula treatment, and psychological or emotional difficulties (e.g., anxiety) due to HCU. Many participants felt that the diet-related and psychological or emotional impacts were especially bothersome. Most patients were also affected by impacts to their education and social lives. Cognition-related symptoms and fatigue were commonly reported and were frequently endorsed as among the symptoms that bothered patients the most. Importantly, many participants described links between adherence to the dietary treatment, their homocysteine levels, and the severity of certain symptoms (e.g., brain fog). Most participants desired a future treatment for HCU that would allow them to take less formula and have a more relaxed diet. Moreover, many believed that even minor changes to the protein restrictions or formula intake would make a meaningful difference in their daily lives.

These findings deepen our knowledge of how patients experience HCU. Many of the diet-related challenges described in this study were similar to those quantified in a survey study of patient and caregiver experiences with HCU (including classical HCU) [5]. On the other hand, the sample in this study reported challenges accessing or paying for treatment more often than the sample in the survey study did (70% vs. 26%) [5]. This could reflect different experiences of patients based in the US vs. those based elsewhere; 100% of the present sample was based in the US, compared to only one-third of the survey sample [5].

Nearly all patients described in the present study were affected by psychological or emotional impacts of HCU, with reports of anxiety and depression being especially common. Similarly, a medical records study of 25 HCU patients noted psychological symptoms in a majority of patients (64%), with a high prevalence of anxiety (32%) and depression (32%) in their sample [9]. The present study provides an in-depth understanding of factors triggering these symptoms, including worries, frustration, and anger about following the diet and worries about current, past, or future physical symptoms.

This study also elicited detailed information about what patients experienced when their homocysteine levels were high. Interestingly, patients described how their symptoms – especially cognition-related symptoms – were worse when they had high homocysteine levels. An association between cognition and homocysteine levels in HCU has been observed previously [10]. In an analysis of 45 patients with HCU, a patient’s homocysteine levels were negatively correlated with their Total Cognition scores on the National Institutes of Health Toolbox Cognition Battery [10].

The symptoms and impacts described in this study should be considered in light of patients’ disease characteristics. For example, half of the patients in this sample considered their homocysteine levels acceptable while the other half felt that their levels were too high. In addition, 80% of patients perceived their HCU to be mild or moderate in severity. Future work in larger samples would be needed to understand how the burdens of HCU are similar or different in patients with homocysteine levels that are controlled vs. uncontrolled and with HCU that is less vs. more severe. Notably, one-third of the sample self-reported that they were Vitamin B6 responsive. Dietary treatment beyond Vitamin B6 supplementation is usually not required in patients who are fully responsive to Vitamin B6 and may be used as an additional treatment in patients who are partially responsive [2, 4]. Patients who respond to Vitamin B6 therapy also generally have a milder form of HCU, even though they may still experience severe complications when not adhering to treatment or if their diagnosis was delayed [4]. In addition, nearly half of the sample was diagnosed via newborn screening. Patients diagnosed as newborns tend to have better outcomes than those diagnosed later [11, 12]. For example, a case-control study of HCU patients found that patients diagnosed through newborn screening were less likely to experience vision problems and had higher IQ scores than those diagnosed later [11]. Further, HCU dietary restrictions can be harder to maintain in patients diagnosed later because they may have already become accustomed to eating certain foods that are not allowed on an HCU diet [3, 4].

Participants in this study were given the opportunity to initiate discussion on any symptoms and impacts that they deemed relevant and to endorse and elaborate on symptoms and impacts prompted by the interviewer. The qualitative interview guide was developed based on a targeted literature review and insights from a patient advocate and three clinical experts, which helped to ensure that the list of prompted concepts was relevant, comprehensive, and understandable. In addition, the study elicited perspectives on the disease experience of both pediatric and adult patients. This may provide insight into symptoms and impacts of HCU at different stages of life. Several concepts (e.g., diet impacts, psychological or emotional impacts, cognition-related symptoms) were widely endorsed across participant groups. However, pediatric patients and caregivers representing pediatric patients endorsed social impacts more frequently than did adult patients, and adult patients endorsed eye or vision symptoms more frequently than did pediatric patients or caregivers.

There are currently gaps in therapy and support for patients with HCU [5]. For instance, patients with HCU may be at risk of malnutrition and micronutrient deficiency [4], especially when they have difficulty accessing methionine-free medical formula and low-protein medical foods or are unable to tolerate the methionine-free formula or recommended vitamin supplements. Findings from this study could be used to guide discussions between patients and their doctors about symptoms and treatments. These findings could also be used to inform the development and evaluation of novel treatments for HCU or to develop patient-reported outcome measures that capture the aspects of HCU that are most meaningful to patients.

This study has several limitations. First, the sample size was small (*n* = 20). However, concept saturation was achieved for both symptom and impact concepts, which suggests that the sample was sufficiently large to elicit key symptoms and impacts of HCU. Larger studies would be needed to assess the generalizability of our findings, to accurately estimate how symptoms or impacts vary between age groups, or to assess how factors such as diagnosis via newborn screening or Vitamin B6 responsiveness may influence patients’ experiences. Another limitation is that the sample included only US-based participants. As such, it is unclear how well these findings represent the experiences of patients living outside of the US. Such patients may have different healthcare systems, different healthcare coverage, and different access to therapies, medical formula, and supplements. In addition, cultural and religious beliefs vary across geographic populations and can affect patient experiences. The authors are aware of numerous anecdotes on the stigma, social hurdles, and other challenges related to having HCU and adhering to the HCU diet in various cultures outside the US. Studies with greater geographic diversity are needed to gain insights into how healthcare, culture, and other factors affect patients’ experiences. Finally, study participants were recruited via a patient advocacy group, which may have led to sampling patients who were more severely impacted by HCU (i.e., those seeking support) and those who have better knowledge and understanding of their disease.

## Conclusions

Many patients were burdened by adhering to a restrictive low-protein dietary treatment and by symptoms that worsened when they were not adherent. More easily managed therapies may improve patients’ everyday lives. These findings can be used to guide clinical care and to help develop treatments that address the symptoms and impacts of HCU that bother patients the most.

## Electronic supplementary material

Below is the link to the electronic supplementary material.

Supplementary Material 1

## Data Availability

The datasets generated and analyzed during the current study are not publicly available due to privacy and commercial restrictions but may be made available by the corresponding author upon reasonable request.
