# Supplementary material for: “My dream is to not have to be on a diet”: a qualitative study on burdens of classical homocystinuria (HCU) from the patient perspective
Source: Orphanet J Rare Dis. 2025 Mar 6;20:106. doi: 10.1186/s13023-025-03576-9 (PMC11884047; doi:10.1186/s13023-025-03576-9)
Supplement: Supplementary file 1 — Supplementary Material 1 [file 13023_2025_3576_MOESM1_ESM.docx]

# Supplementary material

[Supplementary material 1](#_Toc184632808)

[Supplementary methods 2](#_Toc184632809)

[Targeted literature review 2](#_Toc184632810)

[Patient advocate and clinician interviews 4](#_Toc184632811)

[Qualitative interview data collection, coding, and analysis 4](#_Toc184632812)

[Supplementary results 5](#_Toc184632813)

[Signs, symptoms, and monitoring of HCU 5](#_Toc184632814)

[Supplementary Table 1. Signs and symptoms saturation analysis 6](#_Toc184632815)

[Monitoring of homocysteine levels 7](#_Toc184632816)

[Cognition 7](#_Toc184632817)

[Fatigue 7](#_Toc184632818)

[Eye or vision 8](#_Toc184632819)

[Skeletal and dental signs of HCU 8](#_Toc184632820)

[Hair or skin 8](#_Toc184632821)

[Bone 9](#_Toc184632822)

[Vascular or circulatory 9](#_Toc184632823)

[Supplementary Table 2. Representative quotes for the most frequently endorsed signs, symptoms, and monitoring aspects of HCU 10](#_Toc184632824)

[Impacts of HCU 13](#_Toc184632825)

[Supplementary Table 3. Impacts saturation analysis 13](#_Toc184632826)

[References 14](#_Toc184632827)

# Supplementary methods

Prior to the qualitative interviews, a targeted literature review and expert interviews were conducted. The aim of these preparatory steps was to inform and refine the scope of the semi-structured interview guide that was used in the qualitative interviews.

## Targeted literature review

A targeted literature review was conducted to identify signs, symptoms, and impacts of classical homocystinuria (HCU). Searches for relevant manuscripts and conference abstracts were conducted using PubMed/MEDLINE, EMBASE, and bench research approaches (e.g., Google). The Ovid (MEDLINE and EMBASE) search identified 187 manuscripts or conference abstracts (**Supplementary Figure 1**). Of these, 120 references were excluded. Researchers reviewed the full text of 67 documents and then excluded 50 of these references. Following screening, 17 manuscripts or conference abstracts were selected for data extraction. Signs, symptoms, and impacts were also queried from three websites: National Institutes of Health Genetic and Rare Disease Information Center (1), HCU Network America (2), and National Organization for Rare Disorders (3).

Signs, symptoms, and impacts derived from the manuscript abstracts, conference abstracts, and web sources were tabulated and counted according to how many times they appeared in the reviewed sources. Impacts fell into eight categories: diet, treatment burdens, ocular (i.e., eye or vision), bone, cognition, education, psychological or emotional, and psychiatric. Signs and symptoms fell into 11 categories: skeletal and dental signs of HCU, developmental, cognition, homocysteine levels, neurology, ocular, bone, vascular/circulatory, urology, hair and skin, and rare.


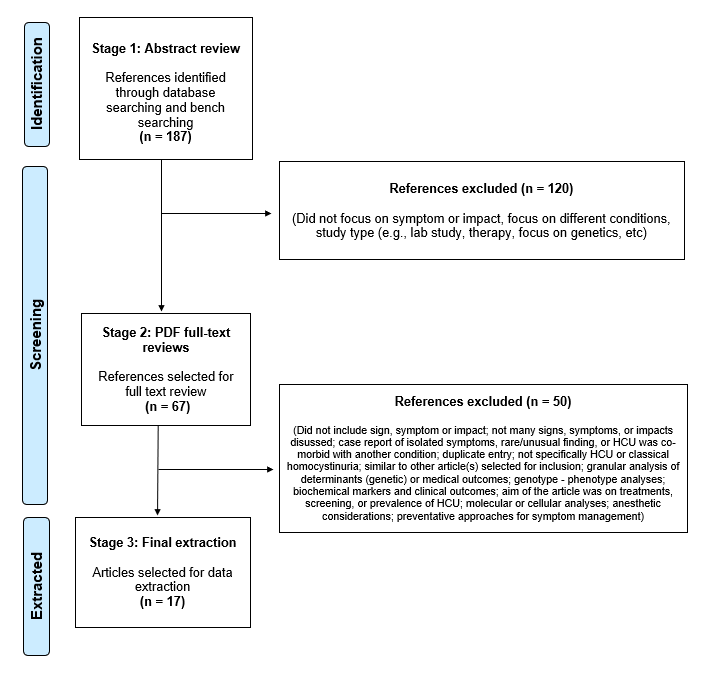


***Supplementary Figure 1. PRISMA flow diagram for the targeted literature review.***

Abbreviations: HCU, homocystinuria; PDF, portable document format; PRISMA, Preferred Reporting Items for Systematic Reviews and Meta-Analyses.

## Patient advocate and clinician interviews

One patient advocate and three clinicians were interviewed about signs, symptoms, and impacts of HCU. Interviews were used to help confirm whether the list of signs, symptoms, and impacts developed from the targeted literature review was comprehensive and accurate. The patient advocate and two of the three clinicians confirmed that the list was comprehensive and accurate. The third clinician commented that urology signs or symptoms were not pertinent or common. Based on these comments, urology was removed from the list of signs or symptoms.

## Qualitative interview data collection, coding, and analysis

Interviews were conducted by experienced scientific researchers who worked for Evidera. Both interviewers were female and had MPH degrees. The interviewers were trained in using the interview guide prior to interviewing participants. At the beginning of the interview, participants were told the interviewer’s name, job title, and that the interviewer was a “research professional”. Participants did not know the interviewer before the study. Only the participant and researcher were present during the interview.

An initial coding dictionary was developed based on the interview guide. The coding dictionary was revised as the transcripts were analyzed and new themes and concepts emerged from the interviews. Three coders attached relevant codes to concepts mentioned within each transcript. Coding was conducted using an iterative coding approach; groups of five transcripts were coded together, and the coding dictionary was updated with new codes before the next group of transcripts was analyzed. To ensure consistency in coding, coders debriefed and reconciled their codes for the first transcript. Queries were escalated to the principal investigator.

Quotes were grouped and summarized by thematic code. Saturation was assessed for the overall sample to quantify when (i.e., at what point) including additional study participants no longer generated any substantially new concepts (4). Participants did not provide feedback on the transcription, coding, or findings, but findings were discussed with the leader of a patient advocacy group (author DB).

# Supplementary results

## Signs, symptoms, and monitoring of HCU

Of the 20 sign, symptom, or monitoring concepts reported, 60% (n=12) were reported in the first five interviews, and an additional 35% (n=7) were reported in the second set of five interviews (**Supplementary Table 1**). Overall, concept saturation for the signs or symptoms was reached at interview 14, after which no new signs or symptoms emerged.

## Supplementary Table 1. Signs and symptoms saturation analysis

| Sign, symptom, or monitoring concept | Participants reporting | |  | Interview group | | | |
| --- | --- | --- | --- | --- | --- | --- | --- |
|  | **n** | **% (n=20)** |  | **1 (n=5)** | **2 (n=5)** | **3 (n=5)** | **4 (n=5)** |
|  |  |  |  |  |  |  |  |
| Vascular or circulatory | 8 | 40 |  | Yes | Yes | Yes | Yes |
| Monitoring of homocysteine levels | 20 | 100 |  | Yes | Yes | Yes | Yes |
| Eye or vision | 10 | 50 |  | Yes | Yes | Yes | Yes |
| Bone | 8 | 40 |  | Yes | Yes | Yes | Yes |
| Cognitive | 15 | 75 |  | Yes | Yes | Yes | Yes |
| Developmental | 5 | 25 |  |  | Yes |  | Yes |
| Neurological | 6 | 30 |  | Yes | Yes |  |  |
| Skeletal and dental signs of HCU | 10 | 50 |  | Yes | Yes | Yes | Yes |
| Hair or skin | 9 | 45 |  | Yes | Yes | Yes | Yes |
| Fatigue | 6 | 30 |  | Yes | Yes | Yes | Yes |
| Stomach problems | 3 | 15 |  |  | Yes | Yes | Yes |
| Weight gain or obesity | 2 | 10 |  | Yes |  | Yes |  |
| Low muscle tone | 2 | 10 |  | Yes | Yes |  |  |
| Respiratory | 2 | 10 |  |  | Yes |  | Yes |
| Pain | 2 | 10 |  |  | Yes | Yes |  |
| Urology | 1 | 5 |  | Yes |  |  |  |
| Sensory issues | 1 | 5 |  |  | Yes |  |  |
| Allergies | 1 | 5 |  |  | Yes |  |  |
| Reaction to meat | 1 | 5 |  |  | Yes |  |  |
| Adenomyosis | 1 | 5 |  |  |  | Yes |  |

“Yes” represents the concept being raised in the interview group. Shading indicates the interview group in which the concept was first recorded.

## Monitoring of homocysteine levels

All participants (n=20, 100%) discussed their homocysteine levels (**Figure 1A; Supplementary Table 2**). Only one participant (5%) – a caregiver – reported that monitoring homocysteine levels was a most-bothersome sign, symptom, or monitoring aspect of HCU.

Many participants discussed how their levels of homocysteine and/or methionine were affected by how well they adhered to dietary treatment. One participant reported that their levels were controlled because they were “very meticulous” about adhering to dietary treatment, while another described not following the diet, struggling with medication, and not seeing progress. One caregiver explained that the doctor recommended lowering their child’s protein allotment to see if that would help reduce homocysteine levels.

## Cognition

Three in four patients (n=15, 75%) experienced cognition-related symptoms due to HCU (**Figure 1A; Supplementary Table 2**). Brain fog was reported across participant groups (adult patients: 5 of 11, 45%; pediatric patients: 2 of 2, 100%; caregivers: 1 of 7, 14%). Several participants explained that their brain fog was linked to high homocysteine levels or not adhering to the dietary treatment.

## Fatigue

Half of patients (n=10, 50%) experienced fatigue (**Figure 1A; Supplementary Table 2**). Some participants reported that the fatigue was more severe when not adhering to the diet; one described getting dressed to be “exhausting” after a few days of not following the diet.

## Eye or vision

Half of participants (n=10, 50%) reported eye or vision symptoms (**Figure 1A; Supplementary Table 2**). Three participants (15%) – all adult patients ­– considered eye or vision symptoms to be among their most bothersome symptoms (**Figure 1B**). Participants experienced divergent trends in their eyesight over time; while a few participants noted that their eyesight issues improved somewhat after diagnosis or after treatment, others reported that their eyesight issues got worse after diagnosis or in adulthood.

## Skeletal and dental signs of HCU

Half of participants (n=10, 50%) reported skeletal and dental signs of HCU (**Figure 1A; Supplementary Table 2**). One participant – an adult patient ­­– considered their skeletal and dental signs of HCU to be among their three most‑bothersome symptoms. Two caregivers attributed their child’s long limbs to poor coordination and balance or awkwardness when engaging in physical activity.

## Hair or skin

Nearly half of participants (n=9, 45%) reported signs or symptoms related to their hair or skin (**Figure 1A; Supplementary Table 2**). Two caregivers described their child’s skin as getting irritated easily or being “itchy a lot”, although the caregivers were unsure whether these skin symptoms were due to HCU.

## Bone

Bone-related symptoms were reported by eight (40%) participants (**Figure 1A; Supplementary Table 2**). Several participants described wearing a back brace or having surgery to help treat their scoliosis, and some explained that they have had “numerous incidences” of broken bones. Arthritis, osteopenia, and osteoporosis were also reported.

## Vascular or circulatory

Eight participants (40%) reported vascular or circulatory symptoms (**Figure 1A; Supplementary Table 2**). Most (4 of 5, 80%) of the adult patients endorsing vascular/circulatory symptoms noted that their symptoms were well maintained with medications and regular check-up visits with their cardiologists. One explained that their hypertension “seems to have been a result of non-compliance with the diet” and “can be managed with diet compliance and exercise”. Several caregivers detailed how serious vascular or circulatory complications ultimately led to their child’s diagnosis with HCU and explained that it took time or several referrals before their child was diagnosed.

## Supplementary Table 2. Representative quotes for the most frequently endorsed signs, symptoms, and monitoring aspects of HCU

| Sign, symptom, or monitoring concept | Example quotes from participant responses |
| --- | --- |
| **Monitoring of homocysteine levels** | “When I was diagnosed, my homocysteine [was] 412. Now with my diet and the medications I'm on and everything, I generally keep my numbers about 25, 26, 27. […] But I'm very meticulous about what I eat and taking all my medications and everything I need to do to keep me in good health.” (Adult patient 300-006-3)  “When we first started this journey, her homocysteine level was over, I think, 1000. And when they immediately put her on the medication, it dropped dramatically. […] Then it's been staggering between, I would say. 173 to 189. […] She was at 42 grams of protein a day. Now we're trying to get that down to see if that correlates. If it doesn't work, then she'll have to take more Vitamin B, B6.” (Caregiver 300-009-2) |
| **Cognitive** | “If they [homocysteine levels] are high, my speech is not clear. I can't think as clearly. Everything's very cloudy.” (Pediatric patient 300-013-1)  “The more elevated [my homocysteine levels] are, the thicker I would say the brain fog is. The two I would say to go hand in hand. […] could just be acting on something like autopilot and kind of like a disassociating.” (Adult patient 300-026-3) |
| **Eye or vision** | “I did have the dislocated lenses. I had both of my lenses removed when I was in my forties also. […] Then I also had retinal detachments in my eyes, too […]. So I had […] reattachment surgery in both of my eyes.” (Adult patient 300-006-3) |
| **Fatigue** | “My energy is lower. I'm always tired to begin with. But my energy is lower, and usually, when I'm bad about my cysteine, I'm bad about my shake. So I get physically fatigued more easily. So when I'm really bad with my diet, […] the act of getting dressed in the morning is exhausting.” (Adult patient 300-024-3)  “[Fatigue] is the one that prevents me from not necessarily doing the things in life but doing less than I want to or should do.” (Pediatric patient 300-013-1) |
| **Skeletal and dental signs of HCU** | “For one thing, I grew incredibly quickly. I was almost the tallest person in my elementary school. I was taller than most of the teachers as well.” (Adult patient 300-024-3) |
| **Hair or skin** | “I actually had really good hair, very thick hair, very long hair and I had a really nice complexion when my levels were good. And when I went off diet, my hair stopped growing. It became very thin, very brittle. And my skin overall tone, looked like kind of grayish, honestly.” (Adult patient 300-005-3) |
| **Bone** | "I wore a back brace from my scoliosis probably for about two and a half years. And I wore that 23 hours a day. I slept in it and everything else.” (Adult patient 300-006-3)  “She tripped and she fractured her toe. For most of us, it would've been a normal ‘Ow, I stubbed my toe’, but for her it was outright fracture.” (Caregiver 300-009-2) |
| **Vascular or circulatory** | “We were there in ICU for a week and they still weren’t sure why she had this clot and strokes […] a month later for follow-up […] she had a bunch more lab work and that’s when they checked her homocysteine level and found it, you know, hugely elevated.” (Caregiver 300-012-2)  “The pediatrician noticed a rather loud heart murmur. […] Then the pediatrician referred us to a cardiologist. And then the cardiologist referred us to the genetic counselor. […] Her blood pressure started to become higher. And so she's now on blood pressure medication for that.” (Caregiver 300-009-2) |

Abbreviation: HCU, classical homocystinuria; ICU, intensive care unit.

## Impacts of HCU

Of the 11 impact concepts reported, 64% (n=7) were reported in the first five interviews, and the remaining 36% (n=4) were reported in the second set of five interviews (**Supplementary Table 3**). Overall, concept saturation for impacts was reached at interview 8, after which no new impacts emerged.

## Supplementary Table 3. Impacts saturation analysis

| Impact | Participants reporting | |  | Interview group | | | |
| --- | --- | --- | --- | --- | --- | --- | --- |
|  | **n** | **% (n=20)** |  | **1 (n=5)** | **2 (n=5)** | **3 (n=5)** | **4 (n=5)** |
|  |  |  |  |  |  |  |  |
| Dietary restrictions and requirements | 19 | 95 |  | Yes | Yes | Yes | Yes |
| Treatment burdens | 17 | 85 |  | Yes | Yes | Yes | Yes |
| Psychological or emotional | 16 | 80 |  | Yes | Yes | Yes | Yes |
| Education | 12 | 60 |  | Yes | Yes | Yes | Yes |
| Psychiatric | 4 | 20 |  |  | Yes | Yes | Yes |
| Social | 11 | 55 |  | Yes | Yes | Yes | Yes |
| Physical activities | 6 | 30 |  | Yes | Yes | Yes | Yes |
| Daily activities | 3 | 15 |  | Yes |  | Yes |  |
| Limited functioning | 2 | 10 |  |  | Yes | Yes |  |
| Sleep | 2 | 10 |  |  | Yes | Yes |  |
| Fatigue | 1 | 5 |  |  | Yes |  |  |

“Yes” represents the concept being raised in the interview group. Shading indicates the interview group in which the concept was first recorded.

# References

1. Genetic and Rare Diseases Information Center. Homocystinuria due to CBS deficiency. 2021. updated February 2023. Available from: <https://rarediseases.info.nih.gov/diseases/6667/homocystinuria-due-to-cbs-deficiency>.

2. HCU Network America. Classical HCU. 2016. Available from: <https://hcunetworkamerica.org/classical-hcu/>.

3. National Organization for Rare Disorders. Homocystinuria due to Cystathionine Beta-Synthase Deficiency. 2021. updated Apr 01, 2021. Available from: <https://rarediseases.org/rare-diseases/homocystinuria-due-to-cystathionine-beta-synthase-deficiency/>.

4. Saunders B, Sim J, Kingstone T, Baker S, Waterfield J, Bartlam B, et al. Saturation in qualitative research: exploring its conceptualization and operationalization. Qual Quant. 2018;52(4):1893-907. <https://doi.org/10.1007/s11135-017-0574-8>.
